# Prevalence of ectoparasitic arthropods on wild animals and cattle in the Las Merindades area (Burgos, Spain)

**DOI:** 10.1051/parasite/2011183251

**Published:** 2011-08-15

**Authors:** G. Domínguez-Peñafiel, C. Giménez-Pardo, M.I. Gegúndez, L. Lledó

**Affiliations:** 1 Consejería de Sanidad y Bienestar Social de la Junta de Castilla y León Spain; 2 Departamento de Microbiología y Parasitología, Universidad de Alcalá Spain

**Keywords:** ectoparasites, wild animals, domestic animals, epidemiology, Spain, ectoparasites, animaux sauvages, animaux domestiques, épidémiologie, Espagne

## Abstract

This paper reports the prevalence of ectoparasitic arthropods in sampled groups of wild (n = 128; 16 species) and domestic (n = 69; 3 species) animals in the Las Merindades area of the Province of Burgos, Spain. The study revealed that wild animals were more infested and with a wider variety of ectoparasites than domestic animals. The parasitic prevalence was 67% for wild animals and 48% for livestock. In this way, 39% of animals were infected by ticks. *Ixodes ricinus* and *Ixodes hexagonus* were the most prevalent species whereas *Dermacentor reticulatus* showed affinity for the fox and wolf. The overall prevalence of parasitisation by fleas was 27%. *Ctenophthalmus* spp. showed the wider range host in wild animals, while *Pulex irritans* was the most frequent specie found. The parasitic prevalences by lice (*Trichodectes melis*, *Trichodectes canis* and *Trichodectes mustelae*) and by mite (*Neotrombicula* spp., *Laelaps agilis* and *Sarcoptes scabiei*) were 4% and 12%, respectively. In both cases only wild animals were found parasited.

## Abstract (fr)

Cet article présente la prévalence des arthropodes ectoparasites dans des échantillons d’animaux sauvages (n = 128 ; 16 espèces) et domestiques (n = 69 ; 3 espèces) dans la région de Las Merindades de la province de Burgos, en Espagne. L’étude a révélé que les mammifères sauvages ont été plus infestés et ont été touchés par une plus grande variété d’ectoparasites que les animaux domestiques. La prévalence parasitaire a été de 67 % pour les animaux sauvages et 48 % pour le bétail. Ainsi, 39 % de tous les animaux ont été infectés par les tiques. *Ixodes ricinus* et *Ixodes hexagonus* ont été les espèces les plus répandues, alors que *Dermacentor reticulatus* a montré une affinité pour le renard et le loup. La prévalence globale du parasitisme par les puces a été de 27 %. *Ctenophtalmus* spp a montré la plus large gamme d’hôtes chez les animaux sauvages, tandis que *Pulex irritans* a été l’espèce la plus fréquemment trouvée. Les prévalences parasitaires par les poux (*Trichodectes melis*, *Trichodectes canis* et *Trichodectes mustelae*) et par les acariens (*Neotrombicula* spp., *Laelaps agilis* et *Sarcoptes scabiei*) ont été de 4 % et 12 %, respectivement. Dans les deux cas, seuls les animaux sauvages ont été trouvés parasités.

## Introduction

Wild animals and arthropods play important roles as zoonotic pathogens reservoirs and/or vectors for humans and domestic animals (Morse, 1995). In the same way, arthropods can transmit a variety of pathogens, such as rickettsias, borrelias, parasitic hemoprotozoa and certain viruses, that cause human diseases and serious infections in livestock. In addition, the behaviour of ectoparasites also may cause direct and indirect harm such as irritation, skin inflammation, pruritus, self-wounding, disturbance and allergic responses (Wall & Shearer, 2001). Few studies have been undertaken in Spain that has jointly examined the ectoparasites that affect both domestic and wild animals. However, the investigations and cataloguing work of Encinas (1986), Domínguez (2004) and Millán *et al*. (2007), among others, have made important contributions to our knowledge of the arthropod ectoparasites of Spain. Even so, the information available on these organisms, their hosts, and the parasitic prevalence is incomplete.

The aim of the present work is to contribute to the knowledge of the distribution of arthropod ectoparasites in the Las Merindades area, in the Province of Burgos, Spain, which lies in the zone of transition between the Cordillera Cantábrica and Sistema Ibérico mountain ranges. The area represents an enclave of varied ecosystems (Román *et al.*, 1996) home to arthropod ectoparasites that could transmit cross diseases to the human, domestic and wild animal populations (Bengis *et al.*, 2004). This work is part of a wider project to improve our knowledge of ectoparasites inhabiting Spain.

## Materials And Methods

### Study Area

The study was performed in the Las Merindades area in the Province of Burgos, Spain (42^o^ 55’ 52’’ N, 3^o^ 29’ 2’’ W). Mean summer temperatures in this area range between 16 and 20 °C, while mean winter temperatures range between 2 and 5 °C. Rainfall is usually high in winter at some 900-1,100 mm/year. The area is mainly rural, but recreational activities attracting non-residents have increased in recent years.

### Animal Samples

197 animals (69 specimens of the three more abundant livestock species and 128 wild animals included into 16 species) were examined for arthropod ectoparasites. Table I shows the composition of these two groups. Not treated livestock came from 20 extensive or semi-extensive farming systems in the study area and were examined during 2007. Wild animals were obtained in different periods and by three methods depending on the species. Small mammals were monthly live-trapped and sacrified later, from 2006 to 2008, except for winter months. Cinegetic species were kindly provided by hunters, during hunting season, between 2005 and 2008. All mustelids and squirrels, and other carnivores, found dead (road kill) during this same period, were included in the study. The sampled animals were combed and examined under magnifying glass, and large animals were explored the entire body surface to recovered ticks, fleas, lice and mites, which were stored in 70 % ethanol in sterile tubes. Identifications were made on the basis of morphometric characteristics using the keys of Beaucornu & Launay (1990) (fleas), Estrada-Peña (2004) (ticks), Martín-Mateo (1977) (louses) and finally Baker *et al*. (1956) and Baker (1999) (mites).

**Table I.** Ectoparasitic arthropods in wild and domestic animals’ prevalence.

		Ticks		Fleas		Lice^+^		Mites		Animals parasitised	
Animals examined (N°)		n	%	n	%	n	%	n	%	n	%
Domestic animals	Cow (35)	12	34							12	34
	Sheep (26)	7	27	15	58					20	77
	Horses (8)	1	13							1	13
	Total domestic animals (69)	20	29	15	22					33	48

Wild animals	Roe deer (12) (*Capreolus capreolus*)	7	58							7^**^	58
	Fox (18) (*Vulpes vulpes*)	15	83	13	72			6	33^S^	17	94
	Wolf (3) (*Canis lupus*)	3	100	2	67	1	33	2	67^S^	3	100
	American mink (25) (*Neovison vison*)	10	40							10	40
	Badger (7) (*Meles meles*)	1	14	4	57	6	86			6	86
	Beech marten (4) (*Martes foina*)	2	50	3	75					4	100
	Pine marten (3) (*Martes martes*)	2	67	1	33					2	67
	Polecat (2) (*Mustela putorius*)	1	50	2	100			1	50* M	2	100
	Stoat(2) (*Mustela erminea*)			2	100	1	50	1	50* T	2	100
	Genet (1) (*Genetta genetta*)	1	100	1	100					1	100
	Wood mouse (26) (*Apodemus sylvaticus*)	11	42	2	8			5	19* T	15	58
	Yellow-necked mouse (8) (*Apodemus flavicollis*)	1	12					3	37^*^^T^	4	50
	Bank vole (6) (*Clethrionomys glareolus*)	2	33	3	50			6	100^T^	6	100
	Red squirrel (5) (*Sciurus vulgaris*)	1	20	2	40					3	60
	Water vole (3) (*Arvicola terrestris*)			1	33					1	33
	Iberian mole (3) (*Talpa occidentalis*)			3	100					3	100
	Total wild animals (128)	57	45	39	30	8	6	24	19	86	67

Total examined animals (197)		77	39	54	27	8	4	24	12	119	60

N°: number of animals examined; n: number parasitised in each category; %: prevalence, expressed as a percentage.

+ Mallophaga;

T mites *Trombiculidae*;

* mites Mesostigmata;

** on sample also with *Hippobosca equina* (Diptera);

S sarcoptic mange (*Sarcoptes scabiei*) in all specimens infested with mites;

M Myobiidae.

## Results

The study revealed that 119/197 (60%) of the animals, 86/128 (67%, CI 95% [59-75%]) of wild animals and 33/69 (48%, CI 95% [35-60%]) of domestic animals, were infested with ectoparasites. Table I shows the hosts of arthropod species. Ticks and fleas were found on 45% (CI 95% [36-54%]) and 30% (CI 95% [22-38%]) of the wild animals, respectively, compared to 29% (CI 95% [18- 40%]) and 22% (CI 95% [12-32%]) of the domestic. Table II (a, b)shows the species of each group of arthropods parasiting on the different hosts.

**Table II a.** Species of ticks identified on the host animals.

Host species (N^o^/Par.)	Animals parasitised per tick species/Prevalence (%)						
*Ixodes ricinus*	*Ixodes hexagonus*	*Ixodes trianguliceps*	*Haemaphysalis punctata*	*Dermacentor reticulates*	*Dermacentor marginatus*	*Riphicephalus turanicus*
Cow (35/12)	12/34			7/20			
Sheep (26/7)				7/27			
Horses (8/1)	1/13						
Roe deer (12/7) (*Capreolus capreolus*)	7/58						
Fox (18/15) (*Vulpes vulpes*)	4/22	7/39		1/5	5/28		1/6
Wolf (3/3) (*Canis lupus*)	1/33				2/67		
American mink (25/10) (*Neovison vison*)		10/40					
Badger (7/1) (*Meles meles*)		1/14				1/25	
Beech marten (4/2) (*Martes foina*)		2/50					
Pine marten (3/2) (*Martes martes*)		2/67					
Polecat (2/1) (*Mustela putorius*)		1/50					
Genet (1/1) (*Genetta genetta*)	1/100						
Wood mouse (26/11) (*Apodemus sylvaticus*)	10/38		1/4				
Yellow-necked mouse (8/1) (*Apodemus flavicollis*)	1/13						
Bank vole (6/2) (*Clethrionomys glareolus*)	1/17		1/17				
Red squirrel (5/1) (*Sciurus vulgaris*)	1/20						

N°: number of animals examined; Par.: number of animals parasitised by ticks.

**Table II b.** Species of fleas identified on the host animals.

	Animals parasitised per flea species/Prevalence (%)							
Host species (N^O^/Par.)	*Pulex* *irritans*	*Ctenoce-* *phalides* *felis*	*Ctenoce-* *phalides* *canis*	*Ctenophthalmus* *spp.*	*Cerato-* *phyllus* *sciurorum*	*Palaeo-* *psylla* *minor*	*Chaeto-* *psylla* *trichosa*	*Paraceras* *melis*
Sheep (26/15)	15/58							
Fox (1S/13) (*Vulpes vulpes*)	7/39	1/6	6/33					
Wolf (3/2) (*Canis lupus*)	2/67							
Badger (7/4) (*Meles meles*)								4/57
Beech marten (4/3) (*Martes foina*)	1/25	2/50					1/25	1/25
Pine marten (3/1) (*Martes martes*)					1/33			
Polecat (2/2) (*Mustela putorius*)				1/50			1/50	
Stoat (2/2) (*Mustela erminea*)				2/100				
Genet (1/1) (*Genetta genetta*)					1/100			
Wood mouse (26/2) (*Apodemus sylvaticus*)				2/8				
Bank vole (6/3) (*Clethrionomys glareolus*)				3/50				
Red squirrel (5/2) (*Sciurus vulgaris*)					2/40			
Iberian mole (3/3) (*Talpa occidentalis*)						3/100		
Water vole (3/1) (*Arvicola terrestris*)				1/33				

N°: number of animals examined; Par.: number of animals parasitised by fleas.

A total of 203 ticks belonging to seven species were collected (Table II a) on 77 samples belonging to the three domestic species examined and 13 of the wild animals. The most prevalent with a wide range of hosts was *Ixodes ricinus*, which was found on 39 animals of ten host species (ungulates, rodents and carnivores). *Ixodes hexagonus* was found on 23 samples belonging to six hosts, all wild carnivores, except for the wolf and genet. Besides, it was the tick that shows the most parasitic intensity on fox, with 51 ticks per animal. *Haemaphysalis punctata*, the third most prevalent tick, was found mainly on livestock, meanwhile *Dermacentor reticulatus* and *Ixodes trianguliceps* were only present on wild canids (fox and wolf) and wild rodents (wood mouse and bank vole), respectively.

A total of 200 fleas belonging to eight species were collected on 54 samples of one domestic species (sheep) and 13 wild host species (Table II b). The most prevalent flea was *Pulex irritans*, which was recovered from 25 animals belonging to three wild carnivores and the sheep, showing the highest intensity of infestation in fox, with 60 fleas per animal. *Ctenophthalmus* spp. was found on nine animals belonging to five wild species, the most range host, three rodents and two mustelids. *Ctenocephalides canis*, *Paraceras melis* and *Palaeopsylla minor* were seen with lower prevalence and with less range of hosts (Table II b).

We also found three species of lice on eight samples belonging to three wild carnivore species, one lice species of Mallophaga per host species. *Trichodectes melis* was observed in six out of seven studied badgers, *Trichodectes mustelae* on a stoat, and *Trichodectes canis* on a wolf (Table III). The intensity of infestation was high in the badgers and stoat, with over 300 lice per animal.

**Table III.** Species of lice (order Mallophaga) identified on the host animals.

Host species (N^o^/Par.)	Animals parasitised per louse species/Prevalence (%)		
	*Trichodectes melis*	*Trichodectes canis*	*Trichodectes mustelae*
Wolf (3/1) (*Canis lupus*)		1/33	
Badger (7/6) (*Meles meles*)	6/86		
Stoat (2/1) (*Mustela erminea*)			1/50

N°: number of animals examined; Par.: number of animals parasitised by lice.

The prevalence of mites among the wild animals examined was 19% (CI 95% [12-26%]). These mites belonged to the species *Neotrombicula* sp., *Laelaps agilis*, *Sarcoptes scabiei* and *Myobia* sp.; these belong to three orders (Astigmata, Prostigmata and Mesostigmata) of the subclass Acari (Table IV). *Neotrombicula* sp. (Prostigmata: Trombiculidae) resulted the most prevalent and was found on twelve animals, while *Myobia* sp. (Prostigmata: Myobiidae) was detected on only one (Table IV). *Sarcoptes scabiei* (Astigmata: Sarcoptidae), the second most prevalent mite, was only seen in the fox (33%, CI 95% [12-55%]) and the wolf (in two out of three exemplars), in which it caused serious sarcoptic mange. *Laelaps agilis* (Mesostigmata: Laelapidae) was seen on wild carnivores and rodents with different prevalences among these species (Table IV). *Neotrombicula* sp. was associated with the highest infestation intensity, reaching some 30 mites per animal in the bank voles.

**Table IV.** Species of mites identified on the host animals.

	Animals parasitised per mite species/Prevalence (%)		
Host species (N^o^/Par.)	Order Prostigmata Family Trombiculidae	Order Mesostigmata Family Laelapidae	Order Astigmata Sarcoptes scabiei
Fox (18/6) (Vulpes vulpes)			6/33
Wolf (3/2) (Canis lupus)			2/67
Polecat (2/1) (Mustela putorius)		1/50^M^	
Stoat (2/1) (Mustela erminea)	1/50	1/50	
Wood mouse (26/5) (Apodemus sylvaticus)	2/S	3/12	
Yellow-necked mouse (8/3) (Apodemus flavicollis)	3/3S	2/25	
Bank vole (6/6) (Cletrionomys glareolus)	6/100		

N°: number of animals examined; Par.: number of animals parasitised by mites; ^M^: simultaneous presence of myobid mites (Fam. Myobiidae).

## Discussion

The mean prevalence of arthropod parasitism in the present sample was higher among the wild than the domestic animals, and among the former it seemed most prevalent between some carnivores. One fox was simultaneously affected by five species: two of ticks, two of fleas, and one of mites. Two of the three wolves examined were infested by three ectoparasites: a flea, a tick and either a louse or a mite species (*Sarcoptes scabiei*). The wolf was the only host to be affected by all the parasite groups. These infestations might be explained by these canids’ shelter, predation and social behaviour.

### Ticks

The most prevalent ticks were *Ixodes ricinus*, followed by *Ixodes hexagonus*, *Haemaphysalis punctata* and *Dermacentor reticulatus*. The hosts with the wide variety of ticks were the fox, and *I. hexagonus* was the most common on them.

*I. ricinus* was the most prevalent and common tick species encountered among all the host species examined, similar to that reported in ectoparasite surveys of the Burgos area by Domínguez (1999, 2004) and comparable regions (Moreno, 1995; Ruiz-Fons *et al.*, 2006). The Las Merindades area provides the ideal conditions for this hygrophilic tick that inhabits forested areas of the northern Iberian Peninsula as well as other, more southerly areas (Travassos Santos Dias, 1994; Manilla, 1998; Habela *et al.*, 2000; Estrada-Peña *et al.*, 2004). *I. ricinus* was the most prevalent tick in human bites in Castilla-León (Fernández, 2003) and also the species most commonly found in cattle in the north area. In this work it was also very prevalent among roe deer (58%), a host species well adapted to this climatical and biological area (Domínguez, 2004).

In other parts of Spain, *I. ricinus* was found on foxes although with a lower prevalence than in the present work (22%): 9% in Doñana (Millán *et al.*, 2007), 12% in Salamanca (Encinas, 1986) and 4.2% in Soria (Serrano, 2004). In the Las Merindades area (Domínguez, 2004), prevalence in foxes of 35% was similar to european studies: in Hungary was recorded as 45% (Sréter *et al.*, 2003), in Germany as 27% (Shöffel et al., 1991) and in France as 33.5% (Aubert, 1975), all countries with a climate similar to the study area. Gilot *et al*. (1976) and Gil (2002) report this tick is very common in northern Spain, especially in wood mice, which are a common host for its immature stages, as founded in small mammals in our area.

*I. hexagonus* was the second species most prevalent, associated with the most intense infestations and nearly 50% of all the ticks collected. This tick species requires endophilic, hygrophilic environments (Travassos Santos Dias, 1994; Hillyard, 1996). It was found on mid-sized carnivores such as the American mink (prevalence 40%; CI 95% [21-59%]), badger, pine marten, beech marten, polecat and the fox (prevalence 39%; CI 95% [16-61%]). In the northeast of Spain this species appears to be absent in foxes (Estrada-Peña *et al.*, 1992), while in Soria it is uncommon (3%; CI 95% [1-5%]) (Serrano, 2004). In Salamanca, it is found in oromediterranean environments, and has been reported in polecats and foxes (Encinas, 1986). In Navarra, it has been cited in the European mink (Díez-Baños *et al.*, 2005). It is not reported in Hungary (Sréter *et al.*, 2003) but appears to be quite prevalent in foxes in other parts of Europe: Germany 18% (Schöffel *et al.*, 1991), France 75.4% (Aubert, 1975) and the UK 33% (Harris & Thompson, 1978).

As reported earlier for the Las Merindades area (Domínguez, 2004), *I. hexagonus* was found in association with *I. ricinus* and *D. reticulatus*, two abundant exophilic species. In southern Spain, these last two species are replaced by *Rhipicephalus* spp. (Encinas, 1986; Millán *et al.*, 2007), but such a substitution is rare in the Las Merindades area, where this genus is not abundant.

*H. punctata* shares habitats with *I. ricinus* throughout its distribution (Manilla, 1998; Estrada-Peña *et al.*, 2004). It is reported very common in northern Spain, both among vegetation (García-Sanmartín *et al.*, 2008) and on cattle and sheep (Moreno, 1995; Domínguez, 1999). However, although it was found in sheep, cow and foxes in the present study, it was not found in great abundance.

The moderately hygrophilic nature of *H. punctata* has allowed it to colonise the northeast of Spain (Estrada- Peña et al., 1992) and Salamanca (Encinas, 1986). In the latter area, it is found in domestic ruminants (prevalence 1.5-10%) and leporids (2%). Millán *et al*. (2007) do not cite it in carnivores in southern Spain, although it does infest other southern hosts (Habela *et al.*, 2000). Ruiz-Fons *et al*. (2006) cite the species to infest red deer in Burgos (which are rare in the present study area), with all inspected animals affected, and in Asturias, where its prevalence in this host falls to 5.5%.

*D. reticulatus* was very prevalent among wild canids and adapted to them (Estrada-Peña *et al.*, 2004), as reported earlier for the Las Merindades area (Domínguez, 1999, 2004) and for Portugal (Santos-Silva *et al.*, 2006). This ectoparasite is active during the colder parts of the year and adapted to the hosts restricted to “green” Spain (Hillyard, 1996; Estrada-Peña *et al.*, 2004). Certainly it is reported absent in foxes from the northeast (Estrada-Peña *et al.*, 1992) and Salamanca (Encinas, 1986), and is uncommon in Soria (prevalence in foxes 0.2%) (Serrano, 2004). Sréter *et al*. (2003) report it in foxes in Hungary with prevalence similar (27%) to that of the present work (28%). It is not found on German (Schöffel *et al.*, 1991) or British foxes (Harris & Thompson, 1978), but does affect the species in France (prevalence 7%) (Aubert, 1975). In the present study area it is one of the ticks that most commonly bite humans (Fernández, 2003).

In the La Rioja region, Zapatero *et al*. (2000) cite *Ixodes trianguliceps* to infest bank voles; in the present study, the prevalence of this parasite in this host was 17%. Gil (2002) reported larvae and nymphs from bank voles, whereas adults were collected in the present work.

*Dermacentor marginatus* and *Rhiphicephalus turanicus* occupy either strictly Mediterranean environments or transition (Travassos Santos Dias, 1994; Estrada-Peña *et al.*, 2004). Estrada-Peña *et al*. (1992) and Encinas (1986) report *D. marginatus* in dry areas, and a prevalence of 7% among cattle in Salamanca. *D. marginatus* is cited in the Las Merindades area in squirrels and hedgehogs, the normal hosts of these ticks when immature, along with small mammals (Domínguez, 2004). Encinas (1986) report its presence in *Apodemus* spp. The present finding of a nymph on a beech marten may have been the result of an accidental infestation. *R. turanicus* is common in the centre-south of the Iberian Peninsula and North Africa (Travassos Santos Dias, 1994; Encinas, 1986; Estrada- Peña et al., 2004), but appears to be uncommon in the study area. It is one of the most important tick of carnivores in Doñana and its surrounding area (Millán *et al.*, 2007), but in the present work was found on a single fox from the southernmost and warmest part of the study area.

### Fleas

We found eight species of flea affecting eight species of wild carnivorous, five of wild rodents/insectivores, and the sheep. Domínguez (2004) recorded 14 flea taxa in 11 mammals species in the Las Merindades area, perhaps reflecting a greater diversity than in southern Spain where Millán *et al*. (2007) recorded only four species on seven wild and domestic carnivorous animals. However, the composition of the sampled animals in these studies was not the same as well as the sampling effort.

In this work, the widest variety of fleas was found on beech martens, with four species. Millán *et al*. (2007) found the same species on the fox in southern Spain. The genus *Ctenophthalmus* was found on the largest number of hosts, five; all wild, followed by *Pulex irritans* which was found on four wild/domestic species.

*P. irritans* can affect a wide range of hosts including humans, although carnivores are preferred (Beaucournu & Launay, 1990; Wall & Shearer, 2001; Domínguez, 2004; Millán *et al.*, 2007). In the present work its prevalence in wolves and foxes was 67% and 39%, respectively. Gil-Collado & Rivas (1976) reported a wolf parasitised by *Ctenocephalides canis*, while Domínguez (2004) observed similar results than the present work on three samples, indicated infestations by *P. irritans* alone. In the south Spain the prevalence of the flea reaches 58% in foxes (Millán *et al.*, 2007), and 67,2% in the same host in Soria (Serrano, 2004). The highest frequency in europen foxes is reported from Hungary (43%) (Sréter *et al.*, 2003); similar to that recorded in the present work but quite distant from the 17.4% observed in France (Beaucournu, 1973; Aubert & Beaucournu, 1976) and the absence in German foxes (Shöffel et al., 1991). Although in Spanish dogs its prevalence is just 1.4% (Gracia *et al.*, 2007), it can develop plagues in optimal enviromental conditions, such recorded Gracia *et al*. (2000) in dogs (prevalence 100%) in certain pounds.

In the present work, *P. irritans* was an important ectoparasite of sheep, although in other areas prevalences of 100% have been reported (Gracia *et al.*, 1999). As indicated by the later authors, the sheep would appear to be this flea’s main host. This adaptation might, however, be recent, since earlier authors suggested the sheep not to be a good host for this species (Urquhart *et al.*, 1985).

The genus *Ctenophthalmus* affected all the fleaparasitised bank voles detected. Its prevalence in wood mice, however, was low at 8%, similar to that reported for Galicia by Pereira *et al*. (1987). Although it is cited in rodents in Spain (Cordero del Campillo et al., 1994; Beaucournu & Launay, 1990) it has not been reported in carnivores, except for the stoat and wild cat (Domínguez, 2004). In the present work, it was found in the polecat and stoat. It has also been reported in stoat in France (Beaucournu, 1973). The wide host range of the species seen in the present work, whether accidental or primary, was the most eclectic of all those recorded.

*Ceratophyllus sciurorum* was found on the red squirrel, its main host according to Beaucournu & Launay (1990), in the study area. Similar data were previously reported by Domínguez (2004). However, the results show than the flea commonly affect treedwelling carnivores, perhaps through the predation of squirrels or the occupation of their nests. The species was found on one of the three pine martens and the single genet examined; these both mustelids prey on squirrels and edible dormice. Domínguez (2004) reports it to affect wild cats, a predator of similar habits. In France, Beaucournu (1973) records *C. sciurorum* as a parasite of the polecat, stoat and beech marten, species in which it was not found in the present work.

*Ctenocephalides felis* is well adapted to human environments where can cause public health problems (Beaucournu & Launay, 1990; Wall & Shearer, 2001). While it is the most cosmopolitan of all flea species, it was not found to affect wild animals strongly, being detected in just one fox (prevalence 6%), as cited by Domínguez (2004) for the same area. A similar prevalence has been reported in foxes in Soria (2.2%) (Serrano, 2004) while in southern Spain it was not seen to affect foxes (Millán *et al.*, 2007). In Europe, prevalence founded in foxes is similar: 2% in Germany (Schöffel *et al.*, 1991), 1.2-2.4% in France (Beaucournu, 1973; Aubert & Beaucournu, 1976) and absence in Hungary (Sréter *et al.*, 2003). The french authors cite it in other carnivores, but also with a low prevalence, meanwhile in the present work it is observed in two beech martens, as reported by Domínguez (2004), who also indicated that this flea showed the greatest infestation intensity in this mustelid.

In addition to foxes, *C. canis* parasitizes wolves, dogs (especially feral dogs) and sometimes cats. In this work, in agreement with Domínguez (2004) and Whitaker (2007), *C. canis* would appear to exclusively affect foxes (prevalence in this work 33%). Surprisingly, Millán *et al*. (2007) do not report it in foxes (and report it in only a few lynxes) in southern Spain, while in Soria its prevalence is 16.1% (Serrano, 2004). Beaucournu & Launay (1990) consider its distribution to be irregular. Its prevalence in foxes in Hungary is 11% (Sréter *et al.*, 2003), while in German foxes it is just 1% (Schöffel *et al.*, 1991).

In Spain, the main host of *Paraceras melis* is the badger (Beaucournu & Launay, 1990; Gil-Collado & Rivas, 1976), perhaps almost exclusively according to the present results. Domínguez (2004) reports a prevalence of 71.5% in badgers in the same area, but also reports it to affect five different carnivores, including the wolf and beech marten; indeed, it was the flea with the widest host range in this author’s study. In the present work, it was also seen as an accidental parasite of a beech marten, probably due to the occupation of an abandoned badger sett (a common practice of the beech marten).

*Chaetopsylla trichosa* is a siphonapteran that affects the badger and fox (Beaucournu & Launay, 1990) but in the present work it was found in the polecat and, in agreement with Domínguez (2004), in the beech marten. Once again, its presence in the beech marten is probably owed to this species’ occupation of old badger setts and fox holes. *Palaeopsylla minor* has been reported in subterranean animals such as the mole (prevalence of 80%), and previously on wood mouse in the study area (Domínguez, 2004). In Galicia it affects the same hosts, although prevalences are lower (Pereira *et al.*, 1987).

### Lice

In this area, *Trichodectes melis* is reported to be an important parasite of the badger (prevalence 43%; intensity of parasitisation 300 lice per animal) (Domínguez, 2004). Millán *et al*. (2007), who only examined a few animals, reported 50% to be affected. In the present work, *Trichodectes canis* was detected in wolves, with a prevalence similar to that reported in the first citation of its kind for the area by Domínguez (2004). In addition, *Trichodectes mustelae* was detected for the first time in the stoat, one animal out of two, but with high intensity of parasitisation; until now the only mustelid in which it had been reported was the weasel (Martín-Mateo, 1977).

### Mites

Domínguez (2004) only reported mites (Order Mesostigmata) to affect the polecat (prevalence 14.2%) and mole (50%). These species were studied in the present work, but was parasited by other ectoparasites.

*Neotrombicula* spp. were found in carnivores and rodents. *Neotrombicula autumnalis* has been previously reported with a prevalence of 1.5% in foxes in Soria (Serrano, 2004); the only report of this genus affecting Spanish carnivores. Fernández *et al*. (2001) report this species to bite humans in the Province of Soria, and that it may be a vector of several pathogens. The rodents examined were commonly affected by trombiculids. Indeed, every bank vole examined was affected. Gil (2002) reported a prevalence of 55.5% in bank voles in the Basque Country. The parasitisation intensity of this species was also intense, thus, bank voles may be a good hosts for them.

Mesostigmatans are cited in classic studies on small mammals in Spain (Zapatero *et al.*, 1978; Pereira *et al.*, 1987). However, new hosts for *Laelaps agilis* were found in the present work, including the polecat and stoat (one individual out of two in both cases); their preying on rodents favours their infestation by these mites.

In wolves inhabiting the Las Merindades area, the prevalence of *S. scabiei*, which is responsible for sarcoptic mange, was reported at 67% in the first citation of its kind for Spain (Domínguez *et al.*, 2008). In the few european references available no prevalence is given (see Mörner *et al.*, 2005). The prevalence of sarcoptic mange in foxes of the study area was 33% (CI 95% [14-59%]), near to the 23.1% for the Ebro Valley reported by Gortázar *et al*. (1998). Both datas are larger than the 5.2% reported for the foxes of Soria (Serrano, 2004), and similar from Europe: Hungary 21% (Sréter *et al.*, 2003) and Germany 25% (Schöffel *et al.*, 1991). Mörner (1992) reports prevalences of over 50% in european foxes, but these probably represent the peak of outbreaks. Epidemics of fox sarcoptic mange, depending on the different stains, may pose a threat to wild and livestock animals, and humans. In the latter it causes pseudoscabies.

These studies are important since they provide the data required for producing maps of arthropod ectoparasite distribution, and can then be compared with climatic and biogeographical maps. Many of the ectoparasites recorded in the present work are potential vectors of pathogens to animals and humans (*Rickettsia* spp., *Borrelia* spp., *Babesia* spp. and *Theileria* spp.) and, especially when people venture into the habitats of these organisms. Defining vector species in a particular area is of the foremost importance for disease control. In summary, further studies are must be performed in the same area to determine the vectorial capacity of arthropod species. These data are essential for the development of future control campaigns.
